# Scholasticide and Population Health in the Eastern Mediterranean

**DOI:** 10.34172/ijhpm.10011

**Published:** 2026-05-24

**Authors:** Mohammad Karamouzian, Martin McKee, Ruth Gibson, Karl Blanchet, Fouad Fouad, Roojin Habibi, AliAkbar Haghdoost, Wahid Majrooh, Maziar Moradi-Lakeh, Sameen Siddiqi, Dan Werb, Vahid Yazdi-Feyzabadi, Iman Nuwayhid, Reza Majdzadeh

**Affiliations:** ^1^Centre on Drug Policy Evaluation, MAP Centre for Urban Health Solutions, St. Michael’s Hospital, Toronto, ON, Canada.; ^2^Dalla Lana School of Public Health, University of Toronto, Toronto, ON, Canada.; ^3^HIV/STI Surveillance Research Center, and WHO Collaborating Center for HIV Surveillance, Institute for Futures Studies in Health, Kerman University of Medical Sciences, Kerman, Iran.; ^4^London School of Hygiene and Tropical Medicine, London, UK.; ^5^Stanford University School of Medicine, Palo Alto, CA, USA.; ^6^Geneva Centre of Humanitarian Studies, Faculty of Medicine, University of Geneva, Geneva, Switzerland.; ^7^Liverpool School of Tropical Medicine, Liverpool, UK.; ^8^Faculty of Law, University of Ottawa, ON, Canada.; ^9^Modeling in Health Research Center, Institute for Futures Studies in Health, Kerman University of Medical Sciences, Kerman, Iran.; ^10^Afghanistan Center for Health and Peace Studies (ACHPS), Geneva, Switzerland.; ^11^Institute of Tropical Aquaculture and Fisheries (AKUATROP), Universiti Malaysia Terengganu, Terengganu, Malaysia.; ^12^Department of Community Health Sciences, Aga Khan University, Karachi, Pakistan.; ^13^Division of Infectious Diseases and Global Public Health, University of California San Diego, La Jolla, CA, USA.; ^14^Health Services Management Research Center, Institute for Futures Studies in Health, Kerman University of Medical Sciences, Kerman, Iran.; ^15^Department of Environmental Health, Faculty of Health Sciences, American University of Beirut, Beirut, Lebanon.; ^16^School of Health and Social Care, University of Essex, Colchester, UK.

**Keywords:** Scholasticide, Eastern Mediterranean, Global Health, Academia, Post-Conflict Health

## Abstract

The systematic destruction of academic infrastructure, termed scholasticide, has been normalised across the Eastern Mediterranean Region (EMR) over more than two decades. The doctrine has operated through two coexisting modalities: the targeted assassination of individual scholars and the physical destruction of institutions, escalating in scale from selective killings and partial looting to the dismantling of entire academic systems. Drawing on evidence from several EMR countries, we describe a causal cascade through which acute military strikes and chronic structural exclusion, including sanctions, platform over-compliance, and visa barriers, produce generational health workforce depletion, disrupt core public health functions, and cause population-level health harms. We argue that academic institutions constitute a distinct, upstream structural determinant of population health whose destruction is an attack on health systems, not merely on education. We propose five categories of action, implemented through standing regional and international mechanisms, to protect academic infrastructure across the EMR whenever and wherever it is attacked.

## Introduction

 Universities not only generate knowledge, but also the human capital, systems, and institutional memory that underpin health systems’ resilience. When a faculty of medicine is destroyed, the loss is not one building but decades of future healthcare professionals. When a public health or biomedical research laboratory is bombed, the loss is not just one experiment but an entire node in a disease surveillance network. Yet while the protection of health facilities is well-established in international law, even if this is increasingly violated,^1^ the contribution of academic institutions within health systems is far less recognised.^2^ Their destruction represents a distinct mechanism through which health system failure unfolds, and existing frameworks do not adequately capture it.

 Education is a well-established determinant of health, operating through multiple, interlinked pathways across the life course.^3^ It is also a fundamental human right, enshrined in the United Nations’ International Covenant on Economic, Social and Cultural Rights (Article 13), alongside the right to the highest attainable standard of health (Article 12) and the right to enjoy the benefits of scientific progress and its applications (Article 15(b)).^4^ These rights are structurally interdependent: education is a prerequisite for functioning health systems, while safeguarding health supports access to and continuity of education. The systematic targeting of a nation’s academic infrastructure, a phenomenon termed scholasticide,^5^ encompasses all levels of education and all disciplines, and its consequences extend far beyond health. This editorial focuses specifically on the health consequences of attacks on higher education institutions.

 These consequences arise because universities are fundamentally integrated systems. An attack on a campus destroys not only its explicitly health-related components—medical faculties, research laboratories, and public health programmes—but also the shared infrastructure on which they depend, including libraries, connectivity, administrative systems, and interdisciplinary collaboration. Universities are also core. components of national health research ecosystems, because they train the health workforce, generate clinical and public health knowledge, sustain surveillance systems, and maintain the institutional continuity on which health system governance depends.^2^ The destruction of academic infrastructure is therefore not merely an educational crisis; its consequences manifest as preventable mortality, health workforce depletion, and degraded regional health security.We therefore argue that academic institutions constitute a distinct, upstream structural determinant of population health, whose destruction produces predictable, causal, and measurable threats to health systems, both directly and indirectly, even in the absence of concurrent attacks on healthcare facilities.

###  Scholasticide Across the Eastern Mediterranean

 Since February 28, 2026, the United States (US) and Israeli strikes have destroyed or damaged 32 Iranian universities, killing at least ten professors and 60 students.^6^ On April 2, 2026, strikes severely damaged the Pasteur Institute of Iran, established in 1920 and serving as a World Health Organization (WHO) collaborating centre that has made major contributions to vaccine development, infectious disease surveillance, and public health research across the WHO’s Eastern Mediterranean Region (EMR).^7^ These are not isolated events. They are the latest expression of a doctrine that has become normalised across parts of the region, and insufficiently challenged for over two decades: the premise that a nation’s academic infrastructure constitutes a legitimate military target, justified by vague and, at best, unverified claims about the presence of military facilities within them, their use by military personnel, or, in some cases, without any stated justification.^8-14^ The doctrine of academic expendability has operated through two modalities: the targeted assassination of individual academics and scientists, and the physical destruction or occupation of institutions, both of which have coexisted across the EMR. What has escalated is their scale and completeness; from the selective killing of individual knowledge holders and the partial looting of campuses, to the systematic dismantling of entire institutional systems, including the equipment, samples, knowledge resources, and organisational capacity that sustain them.

 In Iraq, over 430 academics were killed and universities were systematically looted, burnt, or occupied following the 2003 US-led invasion, and attacks on higher education have continued, with professors targeted through 2023, devastating a generation of Iraqi scholarship without prompting significant international accountability.^10,15^ In the occupied Palestinian territory, where Karma Nabulsi first coined the term “scholasticide” in 2009 to describe systematic targeting of educational institutions,^5^ Israeli forces have repeatedly raided, tear-gassed, and forcibly entered West Bank universities,^16^ while in Gaza, all universities have been destroyed,^17^ with at least 1270 university students and over 95 university professors killed since October 2023.^18,19^ In Lebanon, Israeli airstrikes in March 2026 struck the Rafik Hariri Campus of the Lebanese University in Hadath, killing senior academics in an outdoor courtyard.^20^ Moreover, mass displacement across the country (over 1.1 million people) has severed students, staff, and faculty from their academic institutions, particularly throughout southern Lebanon.^21^

 The perpetrators across the region vary, including government forces, foreign coalitions, and non-state armed groups, but the pattern is consistent. In Syria, universities and teaching hospitals were directly targeted, struck by explosions, and repurposed for military use during the country’s civil war.Examples include Aleppo University, where explosions killed at least 87 people in January 2013; al-Kindi University Teaching Hospital, occupied by government forces as a military base and subsequently destroyed in 2013, eliminating both clinical and teaching capacity; and al-Furat University, occupied by armed groups in 2017.^13,22-24^ In Yemen, over 100 universities have been attacked, and several, including Dhamar University in 2014 and Taizz University in 2015, have been occupied by armed forces, disrupting medical and professional training across the country.^25^ In Afghanistan, university campuses were directly assaulted and facilities occupied for military use,^26,27^ with attacks on the American University of Afghanistan in 2016 and Kabul University in 2020 alone killing at least 35 people, including students, faculty, and staff.^28,29^ In Pakistan, attacks on universities have killed at least 30 students and faculty at a single institution, Bacha Khan University, in 2016, while security forces have forcibly disappeared students and used excessive force against campus protests.^30,31^ In Libya, armed groups occupied university facilities, including Sirte University in 2015, and the targeting and abduction of academics and students forced institutional closures during successive phases of civil conflict.^32,33^ In Sudan, universities in Khartoum have been damaged, looted, and occupied by armed forces since the outbreak of civil war in April 2023.^14,34^ Each unchallenged instance of scholasticide lowers the threshold of acceptability for the next. Understanding how such attacks, neither isolated nor anomalous, translate into long-term population health consequences is essential to disrupting this pattern.

###  From Exposure to Outcome

 We use the language of epidemiology as an analogy for what is a product of power politics, because the causal chain, from exposure to outcome, helps identify numerous opportunities for the global community to intervene. While this framework is necessarily reductive and does not capture the full political complexity of these processes, it provides a structured way to trace how upstream exposures translate into downstream health consequences (Figure). These exposures operates at two timescales: time-limited acute military strikes that destroy universities, laboratories, and research centres; and the chronic structural exclusion of academic communities which precedes, compounds, and outlasts the acute phase. The latter includes sanctions that block access to journals, scientific software, and critical research databases; publisher, banking, and platform over-compliance (ie, restricting access beyond what sanctions legally require) that obstructs access to publication systems, professional services, and core research infrastructure even for routine scholarly activity^35^; and visa regimes that render international fellowship offers unattainable.^36-38^ These constraints intensify when conflict or government responses to civil unrest disrupt electricity, internet connectivity, and telecommunications, severing the channels through which manuscript submission, data exchange, remote supervision, and international collaboration ordinarily continue even under conditions of physical insecurity.^35,39^ The cascade of harm unfolds over years and decades. Iraq’s academic system, for instance, was devastated after 2003; two decades later, its research output, training capacity, and institutional continuity have not fully recovered, and the resulting brain drain is among the largest in the region’s modern history.^40,41^

**Figure:**
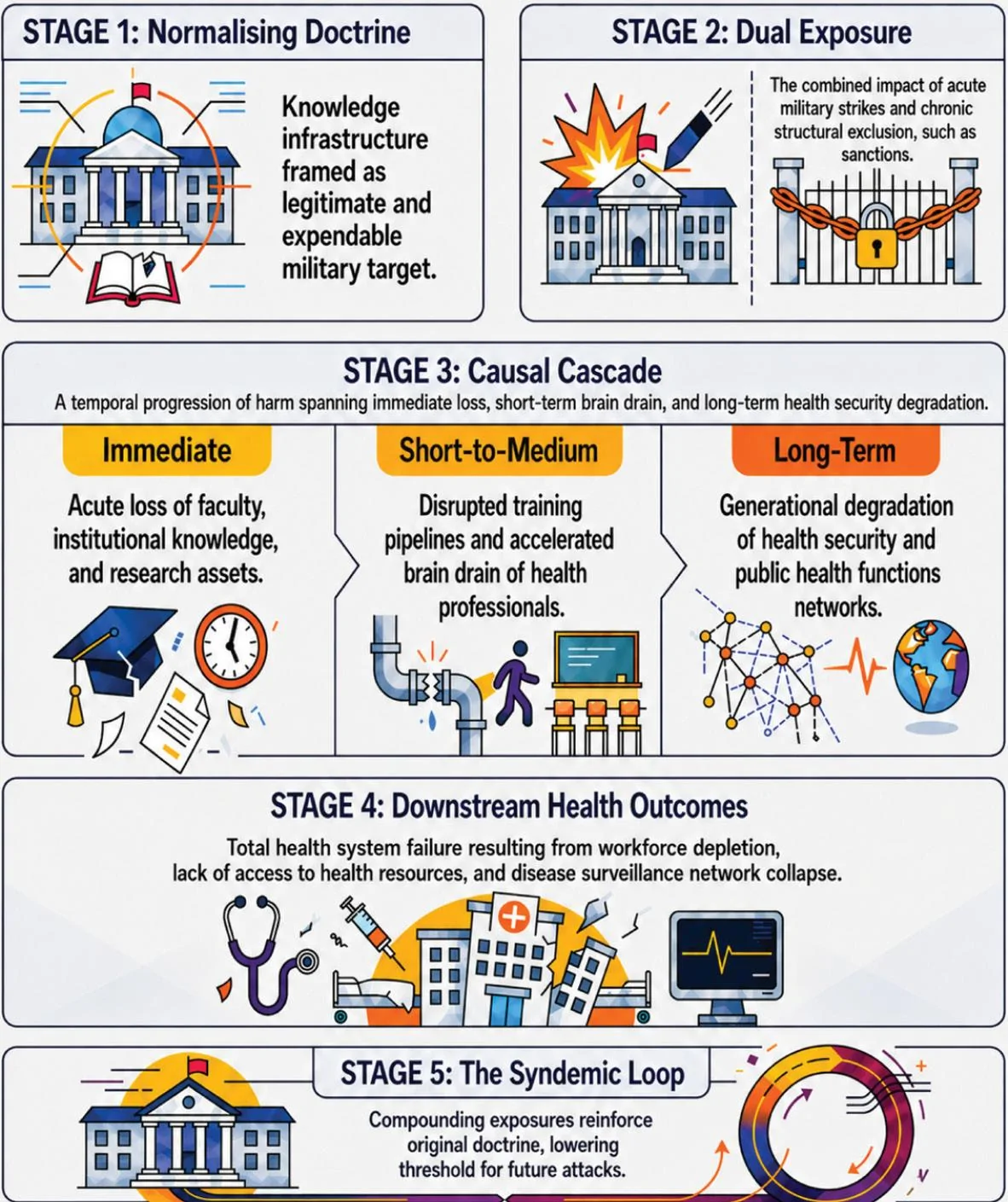
The Cascading Consequences of Scholasticide: From Doctrine to Health System Collapse.

 The mechanisms are consistent across cases. Destroyed laboratories eliminate more than physical infrastructure: they erase ongoing research programmes, specimen collections, institutional knowledge, supervisory capacity, organisational memory, and cumulative routines that cannot be reconstructed from publications alone. Disrupted training pipelines deplete the health workforce, undermining not only immediate capacity but the retention of skilled researchers and professionals on which future recovery depends.^2^ Brain drain accelerates as degrees from affected institutions lose international credibility, creating a paradox in which emigration becomes professionally necessary but personally impossible. Over the long term, the generational consequences consolidate. The Pasteur Institute of Iran, for instance, which has anchored regional vaccine development, disease surveillance, and public health research capacity, illustrates this convergence: its physical destruction could disrupt key public health functions across the EMR, while sanctions and platform exclusion block its surviving researchers from accessing the databases and networks they would need to rebuild. Research programmes that took decades to build cannot be reconstituted in years.

 These structural exposures are deeply intertwined. Sanctions and platform exclusion are mechanisms of economic coercion,^42^ compounding military destruction and preventing the recovery that might otherwise begin between conflicts. When combined with interruptions to communication infrastructure, these pressures produce a condition of scientific isolation in which researchers may remain physically present yet progressively disconnected from the exchanges, transactions, and institutional routines that sustain academic life.^35,43^ Each turn of this cycle does more than cause immediate damage; it normalises the doctrine that academic infrastructure is expendable and targetable, lowering the threshold for the next instance. Unlike attacks on healthcare infrastructure, whose devastation is often immediate and visible, attacks on regional academic architecture produce generational health consequences that will outlast ceasefires and even peace agreements.

###  Academic Freedom and State Responsibility

 Condemning the destruction of universities in EMR countries does not require defending the governments enmeshed in conflict. Across the EMR, academic freedom has been curtailed by the very states responsible for administering educational systems, through ideological screening, dismissal of dissenting faculty, imprisonment of scholars, disciplinary proceedings against students, surveillance of academic life, and in some documented cases, use of academic and health institutions as instruments of state coercion.^44-48^ These abuses, perpetrated by multiple governments in the region, demand sustained international scrutiny and accountability. They also compound the vulnerability of the very institutions that military strikes then destroy: faculties already depleted by political dismissals have fewer scholars to sustain research programmes; brain drain driven by domestic repression accelerates when strikes begin; and institutions weakened by surveillance and self-censorship have less capacity to rebuild.

 Nonetheless, academic infrastructure, however misused or constrained by the governments that administer it, is part of a nation’s intellectual, cultural, and physical patrimony and, as such, should be understood as belonging to the people, rather than to governments, coalitions, or armed groups that seek to control or destroy it. The scholars dismissed by authoritarian governments and those killed in strikes by foreign forces taught in the same classrooms, supervised the same students, and sustained the same institutions. Neither type of violation justifies the other; both are symptomatic of the same forces of structural violence. Those who hold power will eventually leave office, sign ceasefires, or be overthrown; the communities that depend on these institutions have no such exit. They remain, inheriting the consequences.

###  Legal Protections

 International humanitarian law (IHL) provides the normative framework for interrupting this cycle. This includes treaty provisions that bind governments that have ratified the corresponding instruments or are acting on the territory of a state that has done so, as well as the concept of universal jurisdiction under customary international law and certain conventions, including those prohibiting torture and genocide. Additional Protocol I of the Geneva Conventions, although not ratified by the US, Israel, or Iran, explicitly protects civilian objects (Art. 52), which include schools, and by extension, universities, unless and for such time as they become military objectives.^49^ The Rome Statute, ratified by Palestine but not by the three aforementioned countries, classifies intentional attacks on buildings dedicated to education as war crimes under Articles 8(2)(b)(ix) and 8(2)(e)(iv), provided they are not military objectives.^49,50^ Even where countries have not ratified these instruments, intentionally attacking schools and universities in their civilian function remains unlawful under customary IHL.^51^

 In practice, parties responsible for these attacks often invoke the concept of dual-use (ie, the claim that a civilian facility also serves a military function, thereby rendering it a legitimate target) to justify an expanding scope of attacks on civilian infrastructure. However, this doctrinal expansion has been described by legal scholars as one of the most dangerous developments in contemporary IHL.^52^ Under IHL, an object is either a military objective or it is not, and attackers are required to distinguish any military objective from surrounding civilian infrastructure.^53^ Failure to make this distinction can itself constitute a violation.^53^ Over 100 US-based legal experts have condemned strikes on Iranian civilian infrastructure as potential violations of IHL and called for an independent investigation.^54^ The United Nations Educational, Scientific and Cultural Organization has also unequivocally condemned attacks on educational institutions.^49^ These protections must be applied consistently to all actors, in all conflicts, across the entire region.

###  A Framework for Action

 The most direct intervention is, of course, the cessation of attacks on academic infrastructure, a legal obligation, not a request. Pending compliance with IHL, the global health community must act at every other point in the causal chain, and it must do so not only for the ongoing regional war, but through standing mechanisms that protect academic infrastructure across the EMR whenever and wherever it is attacked. Among the most urgent needs is the preservation of functional channels through which scientific work can continue despite conflict, sanctions, and administrative over-compliance. Access to communication systems, journals, databases, software, payment mechanisms, and cross-border academic exchange should be treated as part of an emergency architecture for continuity, rather than as peripheral technical concerns.^35,55^

 Drawing on precedents from prior crises and the documented experiences of scholars in conflict-affected and sanctioned settings, we propose five broad categories of action, mapped to the stages of the cascade described above: (*i*) documenting the exposure through expanded surveillance systems, including a WHO EMRO (Regional Office for the Eastern Mediterranean) tracker for attacks on academic health infrastructure and a standing United Nations mandate to monitor, report, and support accountability processes on scholasticide across the EMR (immediately actionable); (*ii*) removing the structural barriers that compound military destruction,^35^ including clarifications from the Office of Foreign Assets Control (OFAC) and the European Union (EU) clarification that academic publishing, data sharing, and scholarly collaboration are exempt from sanctions; explicit guidance from editorial governing bodies (eg, International Committee of Medical Journal Editors [ICMJE], Committee on Publication Ethics [COPE]) confirming that reviewing and publishing work from sanctioned countries is not a sanctionable activity; and maintaining access to communication, collaboration, and online learning platforms by technology providers and publishers for scholars in conflict-affected and sanctioned settings (requires policy change; precedents exist); (*iii*) sustaining the displaced research workforce through emergency funding and scholar rescue mechanisms, including grants for displaced EMR scholars and accelerated visa processing for academics (requires coordination; modelled on responses to the Russian reinvasion of Ukraine in 2022); (*iv*) protecting the training pipeline through proactive guidance on accreditation and degree recognition, institutional twinning, and emergency cross-enrolment and mutual support among surviving campuses within affected countries (immediately actionable by accreditation bodies, professional societies, and institutional leadership); and (*v*) enabling individual researchers and affected institutions to act now, through co-supervision, preprint co-sponsorship, data sharing, virtual clinical teaching, peer review, and institutional advocacy (Table).

**Table:** Stakeholder-Specific Actions to Protect Academic Infrastructure in the Eastern Mediterranean Region*

**Stakeholder**	**Proposed Action(s)**	**Mechanism/Precedent**	**Feasibility**
UN Agencies	Expand the WHO’s SSA to document strikes on academic/research institutions with health functions across the EMR; assess cascading regional impacts on vaccine supply, disease surveillance, and cross-border health services.	SSA is already operational in EMR; its scope could be expanded and applied retroactively to prior EMR cases.	Immediately actionable.
UN Special Rapporteur on the Right to Education/UNHRC	Investigate and document each targeted facility’s civilian function across the EMR; assess whether previous non-accountability created permissive conditions for subsequent attacks.	Existing mandate holder; independent of the Security Council; compliance assessment under Rome Statute Articles 8(2)(b)(ix) and (e)(iv), and ICESCR Article 13.	Immediately actionable.
Sanctions-imposing governments (eg, US/EU/UK)	Issue standing humanitarian exemptions for academic collaboration in conflict-affected EMR states, covering journals, software, conferences, and data-sharing; provide sufficiently clear compliance guidance to reduce the practical exclusion created by institutional and financial over-compliance.	OFAC General License D-2 provides a relevant model; EU and UK frameworks could mirror this approach.	Requires policy change; precedent exists.
International funding bodies (eg, Wellcome Trust, IDRC, Aga Khan, European councils)	Establish standing EMR Academic Emergency Fund: 12–24-month bridge grants for equipment, data recovery, salary support; pre-authorised for activation upon GCPEA/SAR documentation of systematic attacks.	Modelled on Wellcome Trust’s Ukraine fund and the ERA4Ukraine portal, US-based funders (eg, NIH) could advocate for OFAC general licence authorising academic collaboration with scholars from sanctioned countries.	Requires coordination and funding; precedent exists.
Academic networks (eg, SAR, IIE SRF, CARA) + governments	Issue emergency placement calls for EMR academics; audit unresolved placements from affected settings; publish an annual EMR Scholasticide Monitor tracking fellowship-visa gap.	Afghan 2021 and Ukraine 2022 placement calls provide precedents; Canada’s Special Immigration Measures Programme provides a visa fast-tracking model; UK, EU, and Australian equivalents are needed.	Placements are actionable; visa reform requires government action.
Medical and scientific journals	Fast-track submissions; waive APCs; maintain emergency access to submission systems and core research resources for scholars in conflict-affected and sanctioned settings; review access restrictions that interrupt legitimate scholarly participation; call on editorial governing bodies (ICMJE, COPE, WAME) to issue guidance clarifying that reviewing and publishing academic work are not sanctionable activities; commission peer-reviewed assessments of training pipeline and public health impacts.	Multiple precedents exist from COVID-19 and Ukraine; addresses structural publication inequity affecting all EMR conflict states; journals currently over-comply due to legal uncertainty—governing body clarification removes this barrier without requiring legislative change.	Immediately actionable.
Information technology and online learning platforms (eg, Microsoft, Google, GitHub, Coursera, cloud computing providers)	Maintain access to communication, collaboration, and online learning tools for scholars in conflict-affected and sanctioned settings; resist geo-blocking of routine academic use; provide emergency free-tier access to cloud storage, computing, and virtual classroom infrastructure; issue public policies clarifying that academic use is maintained regardless of sanctions status.	Microsoft and Google provided emergency access to Ukrainian institutions in 2022; OFAC’s informational materials exemption already covers many digital communications; several platforms distinguish between government and individual users in sanctioned countries.	Requires policy change by platforms; precedents exist.
Professional societies (eg, WMA, IAU, national associations, academies)	Joint cross-organisational statements naming affected institutions and responsible actors regardless of identity or alliance; establish standing twinning arrangements for curriculum continuity, remote teaching, and shared examinations.	Twinning modelled on existing academic partnerships; statements must apply the same standard to all actors for credibility.	Statements are immediately actionable; twinning requires institutional coordination.
Accreditation bodies (eg, WFME, ECFMG)	Issue proactive, region-wide, standing guidance confirming degree recognition for all conflict-affected EMR states; establish alternative completion arrangements for interrupted training.	Must be issued before a crisis point to prevent accreditation uncertainty from driving additional brain drain.	Immediately actionable.
Non-affected institutions (eg, surviving campuses across EMR conflict states)	Activate emergency cross-enrolment protocols for displaced students from damaged institutions; establish temporary shared faculty arrangements to maintain teaching continuity; preserve and digitise research data, specimen records, and institutional archives at risk of destruction.	Cross-enrolment of displaced students by Iraqi universities after 2003 and by Afghan, Syrian, and Palestinian universities during prior conflicts.	Immediately actionable by institutional leadership; may require institutional coordination and flexibility.
Individual researchers	Remote co-supervision and examination for displaced students and early-career researchers; co-sponsor preprint deposition for colleagues blocked from journal platforms; share datasets, software licences, and computing access; peer review manuscripts from affected institutions; support stranded faculty in delivering virtual clinical teaching; advocate within home institutions for visiting positions, tuition waivers, and MOUs.	Actionable by individual researchers and departments without centralised approval; distributed academic infrastructure is resilient to strikes and sanctions, as modelled by COVID-19 remote teaching and diaspora-led clinical education initiatives.	Immediately actionable.

Abbreviations: UN, United Nations; APCs, article processing charges; CARA, Council for At-Risk Academics; COPE, Committee on Publication Ethics; ECFMG, Educational Commission for Foreign Medical Graduates; EMR, Eastern Mediterranean Region; ERA4Ukraine, European Research Area for Ukraine; EU, European Union; GCPEA, Global Coalition to Protect Education from Attack; IAU, International Association of Universities; ICESCR, International Covenant on Economic, Social and Cultural Rights; ICMJE, International Committee of Medical Journal Editors; IDRC, International Development Research Centre; IIE SRF, Institute of International Education Scholar Rescue Fund; MOU, memorandum of understanding; NIH, National Institutes of Health; OFAC, Office of Foreign Assets Control; SAR, Scholars at Risk; SSA, Surveillance System for Attacks on Health Care; UNHRC, United Nations Human Rights Council; WAME, World Association of Medical Editors; WFME, World Federation for Medical Education; WHO, World Health Organization; WMA, World Medical Association. * Note: These proposals are intended as a framework for action, not a finalised plan. They draw on documented precedents from prior crises and the experiences of scholars in conflict-affected and sanctioned settings. Their implementation should be developed in consultation with affected academic communities across the EMR, whose expertise and priorities must shape the operational detail.

 History teaches that silence in the face of violations of IHL is not prudence; it is precedent. Each unanswered instance of scholasticide normalises the doctrine that academic infrastructure is a legitimate target of war. From Iraq’s assassinated professors to Gaza’s obliterated universities to Iran’s burning laboratories, the pattern is clear and accelerating. For these protections to retain meaning, condemnation must be translated into measurable follow-through: transparent reporting, independent review, and accountability for perpetrators, as well as scrutiny of institutional responses. If the global health community does not interrupt this cycle now, through standing international mechanisms rather than one-off crisis responses, we risk forfeiting the moral authority to invoke these protections in any future conflicts, for any people. Human rights, including the rights to education, science, and health, are not negotiable. Safeguarding them is our collective responsibility. That responsibility demands not only condemnation, but specificity, consistency, and concrete action.

## Disclosure of artificial intelligence (AI) use

 During the preparation of this work, the authors used Grammarly to check spelling, grammar, and punctuation, and Google NotebookLM to support figure creation. The authors reviewed and edited the content as needed and take full responsibility for the content of the publication.

## Ethical issues

 Not applicable.

## Conflicts of interest

 Authors declare that they have no conflicts of interest.

## References

[1] Kaplan J (2022). Targeting healthcare in war: a tragically tried and tested strategy that humanity must disown-an essay by Jonathan Kaplan. BMJ.

[2] Rasanathan K, Compaoré R, Chopra M (2026). Tackling the global health research financing emergency to sustain national health research ecosystems. Lancet.

[3] The Lancet Public Health (2020). Education: a neglected social determinant of health. Lancet Public Health.

[4] United Nations. International Covenant on Economic, Social and Cultural Rights. https://www.ohchr.org/en/instruments-mechanisms/instruments/international-covenant-economic-social-and-cultural-rights. Published 1966. Accessed May 10, 2026.

[5] Desai C, Hammad S, Abu Shaban A, Takriti AR. Scholasticide and resilience: The Gaza Genocide and the struggle for Palestinian higher education. Curriculum Inquiry. 2025. doi: 10.1080/03626784.2025.2558520.

[6] Prager S. Iran Confirms at Least 60 University Students and 10 Professors Killed by US-Israel Bombings. https://www.commondreams.org/news/iran-university-attacks. Published April 15, 2026. Accessed May 10, 2026.

[7] Delshiri A. Healthocide? Why the attack on the Pasteur Institute of Iran is more than a war crime. The New Humanitarian. April 21, 2026. https://www.thenewhumanitarian.org/opinion/2026/04/21/healthocide-why-attack-pasteur-institute-iran-more-war-crime. Accessed May 10, 2026.

[8] Layal D. What we know about the two professors killed in the Lebanese University Hadath strike. L’Orient Today. March 13, 2026. https://today.lorientlejour.com/article/1499031/what-we-know-about-the-two-professors-killed-in-the-lebanese-university-hadath-strike.html. Accessed May 10, 2026.

[9] Stone R. Israeli attack kills two of Iran’s top nuclear weapons scientists. ScienceInsider. June 13, 2025. https://www.science.org/content/article/israeli-attack-kills-two-iran-s-top-nuclear-weapons-scientists. Accessed May 10, 2026.

[10] Adriaensens D. Killing the intellectual class: Academics as targets. In: Baker RW, Ismael ST, Ismael TY, eds. Cultural Cleansing in Iraq. 1st ed. London: Pluto Press; 2010:119-148.

[11] Pratt N. Scholasticide in Gaza: Settler colonial elimination, genocide, and the crisis of academic responsibility. E-International Relations. November 25, 2025. https://www.e-ir.info/2025/11/25/scholasticide-in-gaza-settler-colonial-elimination-genocide-and-the-crisis-of-academic-responsibility. Accessed May 10, 2026.

[12] Mekhennet S, Strobel WP, Tabrizy N, Hudson J. Killing the ‘brain trust’: How Israel targeted Iran’s nuclear scientists. The Washington Post. December 13, 2025. https://www.washingtonpost.com/national-security/2025/12/17/iran-israel-war-nuclear-scientists-frontline-pbs/. Accessed May 10, 2026.

[13] Tozan O (2025). The evolution of the Syrian higher education sector 1918-2022: from a tool of independence to a tool of war. Globalisation, Societies and Education.

[14] Hassan HY (2025). Protect academia in Sudan with global action. Science.

[15] Global Coalition to Protect Education from Attack. Education Under Attack 2024 - Iraq. https://protectingeducation.org/wp-content/uploads/eua_2024_iraq.pdf. Published 2024. Accessed May 10, 2026.

[16] Global Coalition to Protect Education from Attack. Education Under Attack 2024 - Palestine. https://protectingeducation.org/wp-content/uploads/eua_2024_palestine.pdf. Published 2024. Accessed May 10, 2026.

[17] Sondos F. Every University in Gaza Has Been Destroyed. So Have These Students’ Dreams. The Nation. July 26, 2024. https://www.thenation.com/article/world/gaza-students-future/. Accessed May 10, 2026.

[18] UN Office of the High Commissioner for Human Rights. Palestinian children are deprived of education for the third year in a row. Reliefweb website. https://reliefweb.int/report/occupied-palestinian-territory/palestinian-children-are-deprived-education-third-year-row-enar. Published September 16, 2025. Accessed May 10, 2026.

[19] UN Office of the High Commissioner for Human Rights. UN experts deeply concerned over ‘scholasticide’ in Gaza. https://www.ohchr.org/en/press-releases/2024/04/un-experts-deeply-concerned-over-scholasticide-gaza. Published April 18, 2024. Accessed May 10, 2026.

[20] Elgalil TA. Israeli targeted strike on campus kills two academics. University World News. March 15, 2026. https://www.universityworldnews.com/post.php?story=20260315042954614. Accessed May 10, 2026.

[21] United Nations Relief and Works Agency. UNRWA Situation Report #10 on the Lebanon Emergency Response 2026. https://www.unrwa.org/resources/reports/unrwa-situation-report-10-lebanon-emergency-response-2026. Published May 7, 2026. Accessed May 10, 2026.

[22] Global Coalition to Protect Education from Attack. Education Under Attack 2018 - Syria. https://www.refworld.org/reference/annualreport/gcpea/2018/en/122329. Published 2024. Accessed May 10, 2026.

[23] Global Coalition to Protect Education from Attack. Education Under Attack 2024 - Syria. https://protectingeducation.org/wp-content/uploads/eua_2024_syria.pdf. Published 2024. Accessed May 10, 2026.

[24] Syria rebels take back strategic hospital in Aleppo. BBC News. Published December 21, 2013. https://www.bbc.com/news/world-middle-east-25479725. Accessed May 10, 2026.

[25] Global Coalition to Protect Education from Attack. Education Under Attack 2018 - Yemen. https://www.refworld.org/reference/annualreport/gcpea/2018/en/122323. Published 2018. Accessed May 10, 2026.

[26] Global Coalition to Protect Education from Attack. Education Under Attack 2018 - Afghanistan. https://www.refworld.org/reference/annualreport/gcpea/2018/en/122348. Published 2018. Accessed May 10, 2026.

[27] Global Coalition to Protect Education from Attack. Education Under Attack 2024 - Afghanistan. https://protectingeducation.org/wp-content/uploads/eua_2024_afghanistan.pdf. Published 2024. Accessed May 10, 2026.

[28] Matthews D. Afghanistan university attack leaves more than a dozen dead. https://www.timeshighereducation.com/news/afghanistan-university-attack-leaves-more-dozen-dead. Published August 25, 2016. Accessed May 10, 2026.

[29] Kabul University: 22 dead, more wounded as gunmen storm campus. BBC News. November 2, 2020. https://www.bbc.com/news/world-asia-54750839. Accessed May 10, 2026.

[30] Malkin B, Weaver M. Pakistan university attacks: at least 30 dead in terror raid at Bacha Khan University. The Guardian. January 20, 2016. https://www.theguardian.com/world/live/2016/jan/20/pakistan-university-attack-gunmen-storm-bacha-khan-campus-live-updates. Accessed May 10, 2026.

[31] Global Coalition to Protect Education from Attack. Education Under Attack 2024 - Pakistan. https://protectingeducation.org/wp-content/uploads/eua_2024_pakistan.pdf. Published 2024. Accessed May 10, 2026.

[32] Global Coalition to Protect Education from Attack. Education Under Attack 2018 - Libya. https://eua2018.protectingeducation.org/libya. Published 2018. Accessed May 10, 2026.

[33] Global Coalition to Protect Education from Attack. Education Under Attack 2024 - Libya. https://protectingeducation.org/wp-content/uploads/eua_2024_libya.pdf. Published 2024, Accessed May 10, 2026.

[34] Global Coalition to Protect Education from Attack. Education Under Attack 2024 - Sudan. https://protectingeducation.org/wp-content/uploads/eua_2024_sudan.pdf. Published 2024. Accessed May 10, 2026.

[35] Majdzadeh R, Farzanegan MR (2026). Support besieged Iranian scientists. Science.

[36] Yazdi-Feyzabadi V, Zolfagharnasab A, Naghavi S, Behzadi A, Yousefi M, Bazyar M (2024). Direct and indirect effects of economic sanctions on health: a systematic narrative literature review. BMC Public Health.

[37] World Bank. Internationalization of Tertiary Education in the Middle East and North Africa. https://hdl.handle.net/10986/35316. Published March 26, 2020. Accessed May 10, 2026.

[38] Awashreh R (2025). Empowering Arab researchers: overcoming barriers to effective knowledge dissemination. An-Najah University Journal for Research-B (Humanities).

[39] Karamouzian M, Baral SD (2026). Internet shutdowns in Iran and the right to health. Lancet.

[40] Makki I. Higher education in Iraq after 2003: Ongoing challenges. LSE Middle East Centre. October 2023. https://eprints.lse.ac.uk/120575/2/Higher_education_in_Iraq_after_2003_English.pdf. Accessed May 10, 2026.

[41] Miller J, Riendeau C, Rosen J. Lessons in Academic Rescue. An International Higher Education Response in Post-War Iraq. Science and Diplomacy. 2013;2(3).

[42] McKee M, Pagel C, Correia T (2025). A Public Health Response to Economic Warfare. Int J Health Plann Manage.

[43] Bezuidenhout L, Karrar O, Lezaun J, Nobes A (2019). Economic sanctions and academia: Overlooked impact and long-term consequences. PLoS One.

[44] Saliba I. Academic freedom in the MENA region: Universities under siege. European Institute of the Mediterranean. 2018:313-316.

[45] Shojaei S, Taefehshokr N, Ghavami S (2023). Suppression of academics and school training in Iran during the “Woman, Life, Freedom” revolution. Biochem Cell Biol.

[46] Brand LA (2007). Middle East studies and academic freedom: challenges at home and abroad. International Studies Perspectives.

[47] Torture in Syria’s hospitals. Lancet. 2011;378(9803):1606. doi: 10.1016/S0140-6736(11)61682-6 22055030

[48] Dyer O (2024). Israeli doctors participated in torture, alleges released director of al-Shifa Hospital. BMJ.

[49] UNESCO. Convention for the Protection of Cultural Property in the Event of Armed Conflict with Regulations for the Execution of the Convention. https://www.unesco.org/en/legal-affairs/convention-protection-cultural-property-event-armed-conflict-regulations-execution-convention. Published 1954. Accessed May 10, 2026.

[50] International Criminal Court (ICC). Rome Statute of the International Criminal Court. ICC; 2021.

[51] Henckaerts J-M. Customary international humanitarian law: Volume 1, Rules. Vol 1. Cambridge University Press; 2005.

[52] Hathaway OA, Khan A, Revkin MR (2024). The Dangerous Rise of “Dual-Use” Objects in War. Yale LJ.

[53] Human Rights Watch. Questions and Answers: US, Israel, Iran, and the Laws of War. https://www.hrw.org/news/2026/04/08/questions-and-answers-us-israel-iran-and-the-laws-of-war. Published April 8, 2026. Accessed May 10, 2026.

[54] Dannenbaum T, Hamilton R, Haque AA, Hathaway OA, Gabor R. Over 100 International Law Experts Warn: U.S. Strikes on Iran Violate UN Charter and May Be War Crimes. Just Security. April 13, 2026. https://www.justsecurity.org/135423/professors-letter-international-law-iran-war/. Accessed May 10, 2026.

[55] United Nations. Unilateral sanctions threaten scientific research and academic freedom: UN experts. https://www.ohchr.org/en/press-releases/2022/07/unilateral-sanctions-threaten-scientific-research-and-academic-freedom-un. Published July 7, 2022. Accessed May 10, 2026.

